# Classical and Atypical Scrapie in Sheep and Goats. Review on the Etiology, Genetic Factors, Pathogenesis, Diagnosis, and Control Measures of Both Diseases

**DOI:** 10.3390/ani11030691

**Published:** 2021-03-04

**Authors:** Cristina Acín, Rosa Bolea, Marta Monzón, Eva Monleón, Bernardino Moreno, Hicham Filali, Belén Marín, Diego Sola, Marina Betancor, Isabel M. Guijarro, Mirta García, Antonia Vargas, Juan José Badiola

**Affiliations:** Research Centre for TSE and Emerging Transmissible Diseases, Veterinary Faculty C/Miguel Servet 177, Universidad de Zaragoza, 50013 Zaragoza, Spain; rbolea@unizar.es (R.B.); mmonzon@unizar.es (M.M.); emonleon@unizar.es (E.M.); bmoreno@unizar.es (B.M.); hichamfila@gmail.com (H.F.); belenm@unizar.es (B.M.); 683728@unizar.es (D.S.); mbetancorcaro@gmail.com (M.B.); isabelmariagt91@gmail.com (I.M.G.); 648058@unizar.es (M.G.); vargas@unizar.es (A.V.); badiola@unizar.es (J.J.B.)

**Keywords:** classical scrapie, atypical scrapie, prion disease, sheep, goat, review, surveillance, wasting disease

## Abstract

**Simple Summary:**

Classical scrapie is a prionic, neurological, consumptive, and chronic disease that affects naturally domestic small ruminants. It was initially described in the UK, and since then it has spread throughout the world. Atypical scrapie was first diagnosed in Norway in the 1990s, being diagnosed throughout the world since then. Here, we a provide review of scrapie disease, deepening the characteristics of the causal agent, the pathogenesis of the disease, and its transmission mechanisms. We also emphasize the role of genetic factors, the diagnostic techniques, and the surveillance and control methods established in the European Union. Finally, the infectivity of the different tissues is described.

**Abstract:**

Prion diseases, such as scrapie, are neurodegenerative diseases with a fatal outcome, caused by a conformational change of the cellular prion protein (PrP^C^), originating with the pathogenic form (PrP^Sc^). Classical scrapie in small ruminants is the paradigm of prion diseases, as it was the first transmissible spongiform encephalopathy (TSE) described and is the most studied. It is necessary to understand the etiological properties, the relevance of the transmission pathways, the infectivity of the tissues, and how we can improve the detection of the prion protein to encourage detection of the disease. The aim of this review is to perform an overview of classical and atypical scrapie disease in sheep and goats, detailing those special issues of the disease, such as genetic factors, diagnostic procedures, and surveillance approaches carried out in the European Union with the objective of controlling the dissemination of scrapie disease.

## 1. Introduction

Transmissible spongiform encephalopathies (TSE) or prion diseases are a group of neurodegenerative diseases, of fatal outcome, caused by a conformational change of the cellular prion protein (PrP^C^), giving rise to the pathogenic form (PrP^Sc^). Prion diseases affect humans as well as domestic and wild animals, and in total, eighteen different diseases have been described (see Table 1), nine in animals and nine in humans. They are characterized by astrocyte hyperplasia and hypertrophy, spongiform degeneration, and the accumulation of PrP^Sc^, particularly in the central nervous system (CNS).

The first reliable records on TSE data are from the 18th century where classical scrapie was described [1]. This disease, which affects sheep and goats, is characterized by a progressive chronic ataxia that leads to a lack of coordination, intense scratching, and changes in behavior.

In 1987, bovine spongiform encephalopathy (BSE) was first described [2]. It was diagnosed in British cattle that presented scrapie-like neurological signs. At the end of the 1980s, the disease was already considered a serious problem; however, it acquired more importance after its association with the origin of variant Creutzfeldt–Jakob disease (vCJD) [3]. 

Later, in elderly animals, two atypical neuropathological and molecular phenotypes of BSE disease were identified. These atypical strains showed different PrPres (protease-resistant prion protein [PrP 27–30]) features when compared to classical BSE and were classified according to their biological and biochemical characteristics into two groups: type L [4] and type H [5], characterized by the different molecular mass (H-type) of unglycosylated PrPres when compared with BSE. 

One of the possible origins of BSE could be a classical scrapie agent recycled in meat and bone meal generated from infected sheep and goat carcasses, although this has not been reproduced experimentally [6]. Other hypotheses of the origin are the atypical BSE agent, which has been shown experimentally to change into classical BSE after serial passages in wild-type mice [7,8], or the atypical scrapie agent that may convert into classical BSE following serial passages in transgenic mice [9].

Most of the current knowledge regarding TSE in small ruminants has been acquired through the study of the disease in sheep and goats. While it is assumed that BSE in bovine species is not transmitted horizontally, it could do so in small ruminants and behave (with respect to transmission) like scrapie in these species. This review aims to give an overview of classical and atypical scrapie in sheep and goats, with special emphasis on the genetic factors, diagnostic procedures, and surveillance approaches in the European Union (EU) to control the spread of these diseases.

Classical scrapie was the first TSE described more than 270 years ago in sheep in the United Kingdom (UK) [1]. Later, in 1936, its transmissibility was verified for the first time, through the experimental inoculation of healthy animals with the brain and spinal cords of diseased sheep [10], and in 1942, scrapie was described in goats [11]. Like all TSE, it is a lethal, infectious disease that has long incubation periods, usually affecting animals between 2 and 5 years, and, after the appearance of clinical signs, affected animals can survive between 1 and 6 months [12].

Animals affected by this disease begin with behavioral changes, which progress to more obvious neurological signs, such as ataxia, pruritus, hyperesthesia, and cachexia. Clinical signs may vary between affected animals, but the most common behavioral alterations are restlessness, hyperexcitability to external stimuli, resistance to handling, and bruxism, in addition to pruritus, which is a typical sign of classical scrapie and which can cause areas of alopecia by continuous scratching [13].

In 1998, in Norway, an atypical form of scrapie, caused by the Nor98 strain, was detected for the first time; however, it took until 2003 for its official scientific description [14], which was subsequently described throughout Europe and, finally, worldwide [15,16,17]. There are several differences between classical and atypical scrapie regarding their clinical, pathological, biochemical, and epidemiological behaviors (see Scrapie OIE Chapter for a review, [18]). In atypical scrapie, PrP^Sc^ deposits and lesions are mainly found in the cerebellum and not in the obex as is the case in classic scrapie. PrP^Sc^ is not detected in peripheral tissues [19], although infectivity has been demonstrated in the lymphoreticular system (LRS), nerves, and muscles [20].

The origin of classical scrapie is still unclear. There are several records of the disease between the 18th and early 19th centuries, and all agreed scrapie to be a contagious and infectious disease in sheep [21]. The origin of atypical scrapie is also controversial. It is believed to be a sporadic disease and there is currently no evidence for it being infectious. Atypical scrapie can be transmitted to Tg-mice [22] and to sheep [23]; however, there are several features that are not shared with the classical strain, such as infectivity of the placenta, the age of the affected animals, and the mutations of the *PRNP* (prion protein gene) gene that modulate the risk for this disease susceptibility [24].

### Causal Agent

The prion protein is responsible for a group of fatal neurodegenerative diseases that affect both humans and various animal species, both domestic and wild [25]. Hypertrophy and hyperplasia of astrocytes, neuronal loss, neuronal vacuolization in the CNS, and the accumulation of PrPres characterize the main lesions in affected individuals.

Classical scrapie (ovine and caprine spongiform encephalopathy) is the prototype of TSE disease and has been known for a long time—in fact, the first description of this pathology dates from 1732 [26]. All TSE are originated by a transmissible agent with extraordinary properties, such as: (a) induction of long incubation periods (months, years, and even decades); (b) resistance at high temperatures; (c) resistance to formaldehyde treatment; and (d) resistance to ultraviolet and ionizing radiation. All these properties are incompatible with the presence of nucleic acids, which raised suspicions that the causative agent of these diseases lacked genetic material.

Of all the hypotheses raised regarding the etiology of the disease, the only one currently accepted is the one proposed by Prusiner in 1982 that argued that the infectious agent was simply a protein, which he gave the name of prion—proteinaceous infectious particles. This protein-only hypothesis states that the cellular prion protein (PrP^C^) suffers a conformational change and its α-helix-rich folded structure turns into a pathogenic β-sheet-rich conformer (PrP^Sc^) [27]. PrP^Sc^ has abnormal biological properties, such as insolubility and resistance to proteolytic degradation, which confer its pathogenicity. In particular, the etiology of scrapie disease is considered as an acquired form of prion disease, with a high influence of the genetic factors.

Currently, the biological functions of PrP^C^ are unclear; however, and given that its amino acid sequence is highly conserved between species, it is suggested that it may be of special importance in physiological processes. Research described that this protein is capable of smoothing neuronal responses, acting as an intracellular transducer signal, and thus its absence would lead to an increase in neuronal death [28]. It has also been shown that PrP^C^ can function as a cellular receptor for Cu^2+^, assigning it an active role in homeostasis [29].

In addition, several experiments demonstrated that the myelin degeneration phenotype is caused by a deficiency of PrP^C^, which suggests that myelin conservation may represent an important physiological function of PrP^C^ [30]. In fact, there is a natural model of goats lacking the prion protein due to a stop mutation, which caused demyelinating neuropathy and demonstrated the implication of PrP^C^ in peripheral nerve myelin maintenance [31,32]. In addition, the possibility that it is involved in physiological sleep processes has also been indicated [28]. Finally, a recent study claimed that under rare conditions, PrP^C^ refolds to adopt the prion conformation PrP^Sc^ [33], which is likely the reason for the large extension of its incubation period.

Like conventional infectious agents, prions present a wide variety of strains, which can cause different incubation periods, clinical signs, lesions, and deposition profiles of PrP^Sc^. A number of different parameters are used to characterize prion strains:

- Biological properties: prion strains result in specific phenotypes for different diseases, which can be identified by their incubation periods, clinical signs, histopathological lesions (lesion profile), distribution of PrP^Sc^, and the tissue and cellular tropisms that are all studied in mice models.
- Biochemical properties: Each prion strain is associated with a specific group of biochemical characteristics, in order to highlight the stability against denaturing agents, glycosylation patterns, electrophoretic mobility after digestion with proteinase K, and resistance to proteolytic degradation. Research also reported that strains may differ in their binding affinity for copper [34].
- Conformal properties: Different strains can show similar patterns of resistance to protease but can be distinguished by their conformations. The differences in conformation can be revealed by sedimentation techniques [35], light scattering [36], transmission electron microscopy, and atomic force microscopy [37], through studies of structural change, and by circular dichroism [38], by binding staining [39], by site mapping of binding using a conformation-dependent immunoassay (CDI) [40], and, finally, by mass spectrometry [41].

## 2. Genetic Factors

The *PRNP* gene has a variable length, between 16,000 and 22,000 bases, and is made up of two or three exons (depending on the species), although the entire open reading frame (ORF) is in a single exon. The *PRNP* gene has been identified in a large number of species, both domestic and wild. The homology between the different species is very high. A large number of polymorphisms or variants have been described in the *PRNP* gene in different species. The natural development of a TSE is strongly influenced by alterations in the gene host encoding the PrP^C^ protein [42]. These polymorphisms can influence the conversion of PrP^C^ to the pathogenic isoform PrP^Sc^ [43].

### 2.1. Genotype of the PRNP Gene and Classical Scrapie in Sheep

There is a clear influence of the *PRNP* genotype for codons 136, 154, and 171 regarding the susceptibility of classical scrapie. Previous studies identified three polymorphic codons (136 A (Alanine)/V (Valine), 154 R (Arginine)/H (Histidine), and 171 Q (Glutamine)/R/H) in sheep *PRNP* that are related to scrapie resistance/susceptibility status. Alanine, arginine, and arginine at codons 136, 154, and 171, respectively, are associated with protection against classical forms of scrapie. In contrast, PrP variants (VRQ or ARQ) are associated with susceptibility [42]. The main characteristics of the *PRNP* gene and the susceptibility in sheep are:

- The VRQ haplotype is the most closely related to susceptibility to classical scrapie. Homozygous animals for this haplotype are those that present a higher risk. Heterozygotes with the resistant haplotypes (ARR and AHQ) have lower risk.
- The ancestral form ARQ was also associated with susceptibility to classical scrapie, although with a lower risk or less penetration than VRQ.
- The ARR and AHQ alleles were associated with resistance to this TSE, but only if they are present in homozygosis, ARR/ARR or AHQ/AHQ.
- There are some sheep breeds where certain genotypes do not exist or are extremely rare, e.g., VRQ in Suffolk sheep [44] or ARR in Icelandic sheep [45]. In those cases, the susceptibility to the disease is associated with the ARQ haplotype and the resistance to the AHQ haplotype [46].

Dawson and collaborators (1998) [46] highlighted that the interpretation of this classification is based on probabilities and not on certainties. The groupings of genotypes (see Table 2) are based on the risk of developing the disease (R) and have been valued from R1 to R5:

- R1: indicates a very low risk of developing the disease in an individual and a very low risk in the first-generation progeny.
- R2: indicates a low risk to an individual and progeny.
- R3: indicates an individual low risk, but that of the progeny may increase based on the genotype of the other parent.
- R4: indicates that scrapie can be found occasionally and that the progeny has greater risk.
- R5: indicates that this sheep has the highest risk of developing scrapie; protease-resistant prion protein (PrP) where the superscript is the haplotype with three polymorphic codons (136 A (Alanine)/V (Valine), 154 R (Arginine)/H (Histidine), and 171 Q (Glutamine)/R/H).

### 2.2. Genotype of the PRNP Gene and Atypical Sheep Scrapie

In atypical scrapie, the susceptibility is higher in individuals with the AHQ, AHQ/ARQ, and ARR genotypes along with homozygosity for phenylalanine at codon 141 [47].

### 2.3. Genotype of the PRNP Gene and Classical Scrapie in Goats

Studies carried out around the world showed that the caprine species also presents a great genetic variability for the *PRNP* gene. To highlight this, research described that the presence of methionine at codon 142 may represent a lower risk of the individual to develop the disease [48]; in addition, in animals with this polymorphism, longer incubation periods have been observed in certain experimental TSE [49]. Another study carried out in a herd of goats with a high incidence of scrapie showed that animals carrying the I142M mutation had a higher amount of PrP^Sc^ in the brain when compared to animals of an ancestral genotype, although the lymphoreticular system was less affected [50].

Research also described that goats carrying the allele with three octapeptide repeats and simultaneously the W102G mutation showed a low susceptibility to scrapie [51]. In certain Greek goat breeds, research identified that the R143 and H154 variations may offer some protection against natural scrapie [52]. Similarly, a low susceptibility to scrapie was found to be related to R154H and R211Q variations [53,54,55,56], although the R154H polymorphism is considered a risk factor for atypical scrapie [55].

Other polymorphisms, such as N146D and N146S, were found to be related to scrapie resistance in Cyprus goats [57]. In Italy, a study of six scrapie outbreaks found a possible association with resistance to the disease in animals with a glutamine (Q) to lysine (K) mutation at codon 222 [58]. Similar results were described in other herds of scrapie-affected goats in Italy [59] and in France [53]. Other experimental studies with scrapie isolates [60,61,62,63,64] reinforced the hypothesis that Q222K is a protective polymorphism.

The S127 allele has shown some protection against classical scrapie, both in natural and experimental studies [48,64]. The presence of S127 delays the appearance of clinical signs, but not the deposition of the prion protein in the final phase of the disease.

In a study carried out in goats carrying the polymorphisms I142M, R154H, R211Q, and Q222K, infected with natural scrapie orally and intracerebrally, it was confirmed that the R154H and R211Q polymorphisms represent a considerable increase in the resistance to the disease by the oral route, which was even greater than that provided by the I142M polymorphism. In the aforementioned study, Q222K heterozygous individuals and a small proportion of K222 homozygous goats also developed the disease after the intracerebral route, but with incubation periods that were four to five times longer than those of the Q222Q genotype. These results support the point that the K222 variant provides an intense but not protective effect against classical scrapie [63].

To summarize, the most important polymorphisms and those that reinforce the EU to change legislation and implement selection strategies in goats have been described by the European Food Safety Authority (EFSA, [65]).

## 3. Pathogenesis and Transmission of the Disease

### 3.1. Classical Scrapie

In classical scrapie, it is considered that the main source of contamination are the remains of the placenta expelled by infected animals during delivery; however, transmission can also occur vertically or maternally, through the ingestion of milk and colostrum [66] or intrauterine transfer [67,68]. Experimental studies have also indicated that prions can be transmitted through the skin [69,70,71] and aerosols [72,73].

The pathogenic mechanisms that occur in scrapie depend on the strain, the dose, and the route of introduction of the infectious agent, as well as the host genotype [74,75,76,77]. The causative agent penetrates orally into the body through the intestinal tract. However, in experimental studies, other effective routes have been described, such as intracerebral, intraperitoneal, intravascular, intraocular, intranasal, conjunctival, and through scarification of the skin [69,78,79,80,81].

Prions enter primarily through the gut-associated lymphoid tissue (GALT), mostly at the ileal Peyer patches, which act as primary lymphoid tissue. This is why susceptibility to the disease may be greater at early ages [82]. In the “on boarding” mechanism, M cells play an important role and are located adjacent to Peyer’s patches, scattered in the intestinal lining epithelium. Thus, the PrP^Sc^ goes into the lymphoreticular system, where it accumulates and replicates in macrophages and follicular dendritic cells (FDCs) [83].

The palatine tonsil is also considered as an entrance route. After its multiplication in the lymphoreticular system, the agent targets the central nervous system by two main routes: one directly through the peripheral nervous system, without explicit multiplication in the lymphoreticular system, and another that indirectly involves the lymph nodes, spleen, tonsils, and peripheral nervous system [83,84,85]. Finally, it cannot be ruled out that the neuroinvasion occurs partly by the fraction of PrP^Sc^ circulating in the blood [86,87,88]. The first neuroanatomical locations where PrP^Sc^ deposits are detected are the dorsal nucleus of the vagus nerve and the thoracic spinal cord [89,90,91,92].

Epidemiological studies suggest that natural transmission of classic scrapie occurs mainly horizontally, either by direct contact between animals or indirectly through contamination of the environment [93]. Thus, longer contact and exposure time between animals implies an increase in the spread of the disease. Studies estimated that the main sources of environmental contamination in classical scrapie disease are placentas [94,95,96], feces [97], and carcasses of infected animals [98]. Research demonstrated that the prion protein is also eliminated through excretions (feces and urine) or secretions (milk and saliva) [66,97,99,100,101,102,103,104] and semen [105].

Maternal transmission is recognized in sheep classical scrapie under natural conditions, although it is difficult to evaluate, given the possible lateral contagion between animals of all ages. There is some uncertainty regarding the route of infection that is established from a sheep with scrapie to its offspring, and regarding whether the infection occurs in utero, in the postnatal period, or both. The presence of PrP^Sc^ and the infectivity of the placenta, even in preclinical stages of disease [106], suggest that transmission would take place from infected mothers to their offspring during the delivery through the placenta; nevertheless, natural transmission in utero has also been demonstrated [68,107].

### 3.2. Atypical Scrapie

Regarding pathogenesis, atypical scrapie is considered to be a non-contagious form of the disease. PrP^Sc^ is not detected in the peripheral lymphoid tissue; however, infectivity has been demonstrated by bioassay with transgenic mice in the lymphoid tissue, nerves, and muscles [20,108,109]. In the atypical scrapie, the distribution is not primarily focused in the medulla oblongata, but the highest concentration of prion proteins is located in the cerebellum [14].

The rate of infection of atypical scrapie suggests that it could be a spontaneous disease with a genetic influence and the possible participation of environmental and metabolic factors [110]. Within this hypothesis, apparently, there is no risk factor linked to an infectious origin. Regarding its natural potential transmissibility, epidemiological studies showed that the presence of multiple cases of atypical cases in a holding did not preclude the possibility of atypical scrapie being a sporadic disease [111].

## 4. Diagnostic Methods

### 4.1. Clinical Diagnosis

#### 4.1.1. Classical Scrapie

Clinical signs of classical scrapie are generally expected in animals between 2 and 5 years old. This usually affects a small number of sheep of the flock, although acute outbreaks have also been described. It is an insidious disease, and in a large percentage of confirmed cases of scrapie, animals were found dead with no previous signs of the disease [112].

The most common signs of classical scrapie, reviewed by the International Reference Laboratory for TSE [113], are:

- Changes in the mental and behavioral status, such as separation from the flock, bruxism, and repeated licking of the lips.
- Pruritus. A very characteristic sign of classical scrapie, although it is not always present. This pruritus could cause the appearance of alopecia and wool loss. To demonstrate the presence of pruritus, the scratching test can be performed [13,114,115].
- Postural and locomotion changes. Wide-based limb posture, hypermetric movements, and ataxia.
- Head tremors.
- Loss of body condition.

Other less frequent changes include ptyalism and the loss of ruminal fluid through the oral cavity; nystagmus and altered pupillary reflex [116]; hypoesthesia [117]; cardiac and respiratory disorders [117]; compaction of the rumen [118]; and reduction in milk production [119].

A differential diagnosis includes other neurological diseases, as well as those in which there is a loss of body condition and changes in the wool/skin suggestive of pruritus.

#### 4.1.2. Atypical Scrapie

Atypical scrapie is detected mostly in animals older than 5 years [24]. It typically affects one animal per flock, and, due to the fact that most of the affected animals are of slaughtered or fallen stock subpopulations, it is difficult to relate the characteristic clinical signs of the disease. There are some reports that indicate that ataxia is one of the first clinical signs [120]. Other clinical signs observed by farmers include tremors, behavioral and postural changes, and loss of the bodily condition. Abnormal neurological findings are characterized by hind limb ataxia with hypermetria [18]. Visual impairment, the absence of pruritus, and circling have also been observed [18]. Differential diagnosis of the disease in sheep and goats suggests that the lesions are more localized or asymmetrical, which may lead to the diagnosis of suspected listeriosis or a unilateral space-occupying lesion in the brain (tumor, cyst, or abscess) [18].

### 4.2. Laboratory Diagnosis

The official diagnosis of scrapie should be performed in post-mortem CNS tissue samples [18]. In classical scrapie disease, since PrP^Sc^ can be distributed throughout the lymphoreticular system, the detection of this protein in lymphoid tissue, obtained by rectal [121], third eyelid [122], or tonsil [123] biopsy, is the only reliable in vivo diagnostic method for certain genotypes of the *PRNP* gene (see genetic factors above) [19].

#### 4.2.1. Histological Diagnosis: Spongiform Change

##### Classical Scrapie

**Figure 1:**
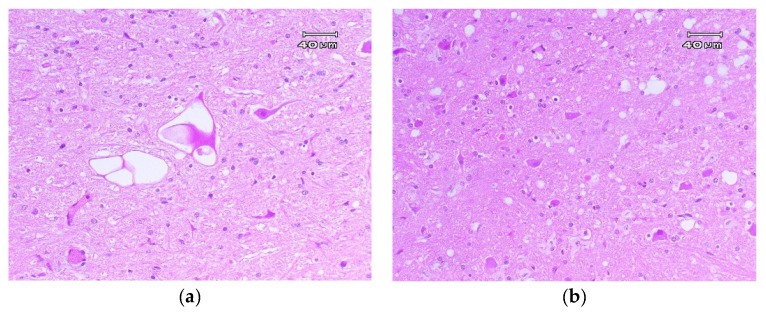
(**a**) Vacuolization of the neuronal perikaryon. Reticular formation. 20×. (**b**) Spongiform degeneration: Vacuolization neuropil grey matter (spongiosis) located in the thalamus. 20×.

The characteristic microscopic lesions of classical scrapie are:

- Spongiform degeneration: Vacuolization, typically bilateral and symmetrical, of the neuronal perikaryon and neuropil gray matter (Figure 1) (spongiosis) located in specific neuroanatomic regions [18,124,125]. The main areas of the CNS where vacuolization is located are the following: In the spinal medulla, the dorsal horns. In the medulla oblongata, the nucleus of the solitary tract, the dorsal nucleus of the vagus nerve, the spinal tract of the trigeminal nerve, the vestibular nuclei, and the reticular formation. In the midbrain, the central gray substance. In the hypothalamus, the paraventricular area, and in the thalamus, the septal area [18,126,127]. The lesion profile can be affected by the strain of scrapie, the genotype of the *PRNP* gene, the pathway of infection, the age of the host, and the duration of the clinical phase [125,128].
- Gliosis. Gliosis is a common and nonspecific response of glial cells against various stimuli and is often present in prion diseases [129]. In scrapie, hypertrophic astrogliosis and an activation of the microglia, generally associated with PrP^Sc^ deposits, vacuolization, and neuronal degeneration, are seen [130,131,132,133]. Both astrocytes and microglia can accumulate PrP^Sc^ in natural and experimental cases of scrapie [95,134].
- Neuronal degeneration and loss. Other characteristic lesions of scrapie are neuronal degeneration and loss, which include disseminated necrotic neurons sometimes accompanied by neuronophagy, dystrophic neurites, and basophilic neurons [135]. Cerebral amyloidosis is also common in scrapie in sheep [127,135,136].

##### Atypical Scrapie

The characteristic microscopic lesions of atypical scrapie also include spongiform degeneration, gliosis, and neuronal degeneration and loss; however, vacuolation in the brainstem is absent and it is more conspicuous in the cerebellar and cerebral cortices [137].

#### 4.2.2. Detection of PrP^Sc^ Scrapie Using Immunohistochemistry Techniques

The immunohistochemistry (IHC) technique detects PrP^Sc^ in situ, allowing for detection of the pathological protein, its distribution in the tissue, its cellular location, and the characteristics of its morphological accumulation [138].

The detection of PrP^Sc^ by IHC techniques allows the diagnosis of scrapie before histopathological lesions are observed in the CNS or when these are minimal or inconclusive [89,90]. Likewise, several studies demonstrated the ability of IHC to detect PrP^Sc^ in tissues where it was not possible to perform a histopathological diagnosis, due to autolysis or freezing [84,139,140,141,142]. PrP^Sc^ deposits can be seen to be associated with histopathological lesions or in areas where there is no vacuolization or where vacuolization is minimal [84,143].

##### Classical Scrapie

**Figure 2:**
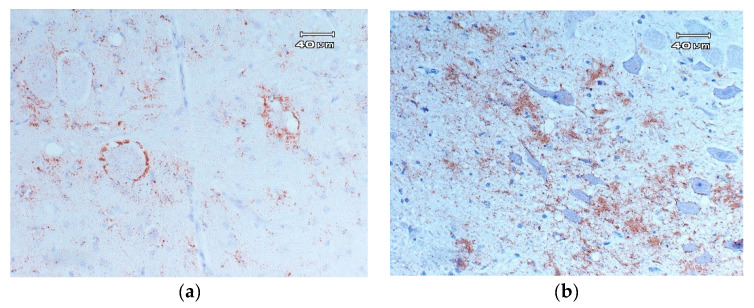
(**a**) PrP^Sc^ intraneuronal and perineuronal immunostaining in neurons located in the brainstem 20×. (**b**) PrP^Sc^ immunostaining in the neuropile of the gray matter located in the brainstem 20×.

Immunostaining is typically bilateral [90] and is located mainly in the brainstem; however, it is often distributed throughout the CNS (Figure 2) [89]. The different types of specific immunostaining of the PrP^Sc^ deposits in scrapie [90,139,143,144] have been described in detail and are characterized by [138,145]:

- Intraneuronal: disseminated granular deposits in the neuronal pericarion.
- Intraglial: disseminated granular or ovoid deposits in the cytoplasm of glial cells.
- Glial-associated: radial deposits associated with glial cells.
- Subpial: multifocal or continuous accumulations, associated with glial cells below the pia mater.
- Perivascular: deposits associated with glia cells but localized around blood vessels.
- Subependymal: deposits, generally discontinuous, located in the glia cell layer located below the ependymal cells of the ventricular system.
- Ependymal: deposits on the apical edge of the ependymal cells.
- Linear: thick deposits in linear organization.
- Fine punctate: small granules in the neuropil.
- Coarse punctate: similar to the previous, but with larger deposits that are irregular in shape.
- Coalescent: deposits in the neuropil that are constituted by the fusion of accumulations of coarse particles of PrP^Sc^, forming amorphous masses, or a mesh-like structure.
- Perineuronal: fine deposits around the neuronal perikaryon and neurites.
- Plaques: large accumulations of PrP^Sc^ with a fibrillary and radiated form, typically localized around blood vessels.

##### Atypical Scrapie

In atypical scrapie, the characteristic gray and white matter PrP^Sc^ deposition is fine punctate to coarse granular [108]. It is not characterized by intraneuronal staining, and deposition in the brainstem is rare; however, the cerebellar and cerebral cortices are generally the most immunostained areas of atypical/Nor98 scrapie brains [108]. The cerebellum is one of the most important areas in atypical scrapie PrP^Sc^ deposition, which has implications for surveillance programs and will be discussed later. Finally, the substantia nigra also showed perineuronal and linear staining in this disease.

#### 4.2.3. Detection of PrP^Sc^ Scrapie Using Western Blotting Techniques

##### Classical Scrapie

Western blotting (WB) is a method to diagnose TSE with high sensitivity and specificity [146]. Both PrP^C^ and PrP^Sc^ have a molecular mass of 33–35 kD [25,147]. When digested with proteinase K, PrP^C^ is completely degraded, while the N-terminal end is removed in PrP^Sc^, leaving a resistant fraction of 27–30 kD, called PrP 27–30 or PrPres [148]. The basic methodology of WB consists of extraction of the resistant fragment of PrP^Sc^ by proteinase K digestion, separation of the proteins in a polyacrylamide gel by electrophoresis, and transfer to a nitrocellulose membrane that is incubated with specific antibodies [149].

##### Atypical Scrapie

The atypical scrapie strain showed, after the previous mentioned proteinase K digestion, a multiple-band pattern. These isolates showed a five-band pattern around 31, 27, 21, 18, and 11 kDa [108].

#### 4.2.4. Diagnosis of Classical and Atypical Scrapie by Rapid Test

The European Commission completed the first evaluation of TSE rapid tests in 1999 [150]. Initially, these evaluations were performed for the diagnosis of BSE, but given the introduction of sheep and goats in the TSE Surveillance Program, they also check for scrapie diagnosis. There are several commercially available rapid tests for use in small ruminant surveillance programs. Following the European reference lab recommendation, the choice of test will be affected by a range of factors including the cost, local availability, and ease of use and/or existing laboratory skill. For more information, the TSE globalnet lab can be consulted [151].

Rapid diagnostic techniques are validated for the detection of the causative agent in the nervous tissue of infected animals. Any technique that aims at the control of these diseases must be scientifically proven and be rigorously validated by the European Union. These techniques allow the diagnosis of a high number of animals in a reasonably short time. In most of these rapid tests, the purification of prion proteins is based on the biochemical differences between the two proteins. Thus, most of them are based on the digestion of PrP^C^ by proteolytic enzymes (proteinase K), and on the resistance of PrP^Sc^ to this enzyme. Finally, all the rapid tests include a step consisting of denaturation of the resistant fraction (PrPres) of PrP^Sc^, in order to allow its binding with the antibody that recognizes PrP^C^ or denatured PrP^Sc^ [152].

Rapid tests identify classical and atypical scrapie indistinctly, but, bearing in mind the differences in PrP^Sc^ deposition, the selection of the sample is absolutely relevant for the confirmation of the disease. In the case of classical scrapie, the brainstem is the most affected area, whereas in the case of atypical scrapie, the cerebellum is typically the most affected.

#### 4.2.5. New Diagnostic Methods of Scrapie

There are some studies that focused on applying new sensitive methodologies to diagnose scrapie. An example is the Real-Time QUaking-Induced Conversion (RT-QuIC) methodology. RT-QuIC is a highly sensitive experimental technique that was first described in 2010 and is characterized by the detection of small amounts of PrP^Sc^ [153]. This technique uses the ability of PrP^Sc^ to induce the misfolding of PrP^C^. It is performed in a cyclical way with the aim of forming aggregates of PrP^Sc^ fibrils. The formation of these aggregates is monitored in real time by binding PrP^Sc^ to the fluorescent dye thioflavin T. In scrapie, it has been used with promising results in recto-anal mucosa-associated lymphoid tissue and in brain homogenates [154].

## 5. Surveillance and Control Methods Established in the European Union

Some measures have been adopted by the European Union with the aim of controlling scrapie disease in sheep and goats. Since the implementation of those control measures, cases of scrapie have constantly decreased in all countries of the EU. Those measures can be summarized below [155]:

- Exclusion of specific risk materials (SRM) from the human food chain. Some studies on the pathogenesis of the disease indicate that infectivity is mainly localized in the CNS and, in the case of scrapie, infectivity is also distributed by the LRS [156]. The tissues with higher infectivity are defined as specific risk materials, and their exclusion from the food chain was one of the most important measures for human consumer protection [157,158,159]. During the bovine spongiform encephalopathy epidemic, sheep and goats were possibly less exposed to contaminated meat and bone meal, which does not exempt the transmission of the disease to these species [160]. In this way, it was shown that small ruminants could be experimentally infected with BSE [161,162,163,164,165], along with diagnosing the first goat BSE case in France by active surveillance [166]. In sheep and goats, the SRM are:
  ○ The spleen and the ileum of sheep of all ages.
  ○ The skull, including the brains and eyes, tonsils, and spinal cord from animals over 12 months or with a permanent incisor erupted.
- Surveillance programs. Surveillance programs of scrapie allow a reliable knowledge of the epidemiological situation in each member state. The annual monitoring program is based on active surveillance (testing without previous suspicion) and passive surveillance (testing of clinical suspects).
  ○ Active surveillance. The active surveillance covers testing of three subpopulations of sheep and goats:
    ▪ Animals over 18 months of age that are not slaughtered for human consumption, such as fallen stock, which have died or been killed, but not in the framework of an epidemic.
    ▪ Animals culled in the framework of TSE eradication.
    ▪ Healthy animals over 18 months of age slaughtered for human consumption. Only member states with major ovine or caprine populations are required to test an annual minimum sample size of such animals.
  ○ Passive surveillance. Testing animals identified as TSE suspects as scrapie is a notifiable disease.
- Genetic selection in sheep. Member states may introduce breeding programs to select for resistance to TSE in their ovine populations. The breeding program shall concentrate on flocks of high genetic merit, as defined in point 3 of Annex I of Commission Decision 2002/1003/EC [167]. In brief, breeding programs are aimed to increase the frequency of the ARR allele; any male animal carrying the VRQ allele shall be slaughtered or castrated. The flocks are finally recognized at two levels:
  ○ Level I flocks shall be flocks composed entirely of ovine animals of the ARR/ARR genotype.
  ○ Level II flocks shall be flocks whose progeny has been sired exclusively by rams of the ARR/ARR genotype.
- Genetic selection in goats. Member states may introduce breeding programs to select for resistance to TSEs in their goat populations. The K222, D146, and S146 alleles confer genetic resistance against classical scrapie strains [57,58,59,168]. Breeding for resistance can be an effective tool for controlling classical scrapie in goats [65].

## 6. Infectivity of the Different Tissues

As has been explained, the term “specific risk material” emerged with the aim of avoiding the risk of transmission of the disease by the consumption of those tissues where scrapie prions replicate. These SRMs are removed from the animal’s carcass during processing in the slaughterhouse. In sheep and goats, the most infective tissues are: the skull (including the brain and eyes), the tonsils, and spinal cords of animals over 12 months of age, as well as the spleen and ileum of sheep and goats of all ages.

Specifically, infectivity has been shown in the tonsils, spleen, and the retropharyngeal, mesenteric, and prescapular lymph nodes. In experimental studies of BSE in sheep, infectivity has been described in these same tissues [169].

The demonstration of blood infectivity in a sheep model, both infected with scrapie or BSE [86,170,171], not only implies the risk of transmission of the disease through this biological fluid, but also increases the risk of spreading the infection to a wide variety of tissues.

Another organ where PrP^Sc^ has been shown is the kidney [101], which could imply the possible impact of the presence of PrP^Sc^ in the urine [172]. According to the reviewed literature, although the infectivity of this fluid has been demonstrated [105], the accumulation of PrP^Sc^ has only been detected in an experimental scrapie model through applying the PMCA technique (protein-misfolding cyclic amplification) [173,174].

Possible infectivity of the muscle has been subjected to intense research due to the importance of this tissue in the human diet. The infectivity of this tissue has been demonstrated in the case of scrapie, not only in experimental models [175,176], but also in a natural model of the disease [177].

Regarding the risk of transmission by semen fluid, the general conclusion to be drawn from the investigation reviewed is that infectivity of semen from infected sheep and goats, although present, seems to be low [105].

In relation to the placenta, the contamination of fields with this organ is one of the most accepted transmission routes in the case of classical scrapie [95,178]. This protein accumulation in the placenta depends not only on the genotype of the mother, but also on the genotype of the fetus [95], being undetectable in the ARR/ARR genotype. A possible role in the transmission from scrapie is also attributed to the saliva. The presence of PrP^Sc^ shown in salivary glands in natural and experimental models of scrapie suggests a potential infectivity of this secretion [102]. In fact, in cervids, the ability to transmit a TSE through this route has been verified by bioassay even in the preclinical stages of the disease [179,180].

The presence of PrP^Sc^ in the mammary glands of co-infected scrapie animals with maedi-visna [181] suggested that milk could serve as a potential transmission vehicle of scrapie. In addition, the presence of PrP^Sc^ in the distal ileum and rectal mucosa from lambs with susceptible genotypes fed with milk from scrapie-affected females demonstrated the transmission of the disease through this route [66,99,100].

## 7. Conclusions

In summary, scrapie is a complex disease that affects small domestic ruminants and is characterized by a peculiar etiological agent (more than 20 strains in classical scrapie and at least one demonstrated atypical one) that behaves differently if polymorphisms in the *PRNP* gene are present. As knowledge advances, new doubts are sown regarding the infectivity of tissues, the transmission mechanisms, and the etiological agent itself. Its infectious character makes this disease difficult to eradicate, and most of the efforts are focused on controlling and preventing the disease with active and passive surveillance as well as the selection of resistant animals to the disease. Prion diseases often evoke a feeling of helplessness because many factors are beyond our control. That is why it is of great interest to continue to reinforce the research in these diseases and obtain achievements in all fields of the disease.

## Figures and Tables

**Table 1:** Animal and human transmissible spongiform encephalopathies (TSE).

**Animals**		
**Disease**	**Host**	**Origin**
Classical Scrapie	Sheep and Goat	Infectious
Atypical Scrapie	Sheep and Goat	Sporadic/spontaneous
Bovine Spongiform Encephalopathy (BSE)	Bovine	Infectious
Atypical BSE L or H	Bovine	Sporadic/spontaneous
Chronic Wasting Disease (CWD)	Deer	Infectious
Feline Spongiform Encephalopathy (FSE)	Feline	Infectious
Transmissible Mink Encephalopathy (TME)	Mink	Infectious
Exotic Ungulate Encephalopathy (EUE)	Antelope	Infectious
TSE in Non-Human Primates	Lemur	Infectious
**Humans**		
**Disease**	**Host**	**Origin**
Iatrogenic Creutzfeldt–Jakob Disease (iCJD)	Human	Infectious
Sporadic Creutzfeldt–Jakob Disease (sCJD)	Human	Spontaneous
Familiar Creutzfeldt–Jakob Disease (fCJD)	Human	Hereditary
Variant Creutzfeldt–Jakob Disease (vCJD)	Human	Infectious
Gerstmann–Sträussler–Scheinker (GSS)	Human	Hereditary
Fatal Familiar Insomnia (FFI)	Human	Hereditary
Fatal Sporadic Insomnia (FSI)	Human	Spontaneous
Kuru	Human	Cannibalism
Variably Protease-Sensitive Prionopathy (VPSPr)	Human	Spontaneous

**Table 2:** Classical scrapie in sheep. Risk groups and genotypes. Adapted from Dawson and collaborators [46].

Genotype	Risk
PrP^ARR^/PrP^ARR^	R1
PrP^ARR^/PrP^AHQ^ PrP^AHQ^/PrP^AHQ^	R2
PrP^ARR^/PrP^ARQ^ PrP^ARR^/PrP^ARH^ PrP^ARQ^/PrP^AHQ^ PrP^AHQ^/PrP^ARH^	R3
PrP^ARH^/PrP^ARH^ PrP^ARQ^/PrP^ARH^ PrP^ARQ^/PrP^ARQ^ PrP^ARR^/PrP^VRQ^ PrP^AHQ^/PrP^VRQ^	R4
PrP^ARQ^/PrP^VRQ^ PrP^ARH^/PrP^VRQ^ PrP^VRQ^/PrP^VRQ^	R5

## Data Availability

Not applicable.
